# Pulmonary Hypertension Among Individuals Living With Hemoglobinopathies: A Systematic Review

**DOI:** 10.7759/cureus.88234

**Published:** 2025-07-18

**Authors:** Collins C Okeke, Angela Ojo, Madeleine O Okere, Onyinye E Ebiliekwe, Ifunanya R Ekeocha, Kris N Idion, Onyeka Egemonye, Omosimisola O Alli, Euodia A Ugo-Ihanetu, Sylvahelen Okorienta, Afamefuna O Onyeogulu, Elo Agidah, Amarachi Nduji, Emmanuel I Akinteye, Olaoluwa E Ebiekuraju, Joseph O Egbunike

**Affiliations:** 1 Internal Medicine, University of Port Harcourt Teaching Hospital, Port Harcourt, NGA; 2 Internal Medicine, Afe Babalola University, Ado-Ekiti, NGA; 3 General Practice, University of Port Harcourt Teaching Hospital, Port Harcourt, NGA; 4 Internal Medicine, Nnamdi Azikiwe University Teaching Hospital, Nnewi, NGA; 5 Internal Medicine, Delta State University Teaching Hospital, Oghara, NGA; 6 Internal Medicine, Nnamdi Azikiwe University College of Health Sciences, Awka, NGA; 7 General Practice, College of Medicine, Lagos State University, Lagos, NGA; 8 Internal Medicine, Rivers State University Teaching Hospital, Port Harcourt, NGA; 9 Public Health, Liberty University, Lynchburg, USA; 10 Internal Medicine, Igbinedion University Teaching Hospital, Okada, NGA; 11 Internal Medicine, Abia State University, Uturu, NGA; 12 Medicine and Surgery, Bowen University, Iwo, NGA; 13 General Surgery, Surgery Interest Group of Africa, Lagos, NGA; 14 Family Medicine, Aeon Medical Center, Abuja, NGA

**Keywords:** hemoglobinopathy, outcome, pulmonary hypertension, risk factor, sickle cell disease (scd), thalassemia

## Abstract

Hemoglobinopathy has a diverse clinical presentation and complications, and is severe among individuals with the homozygous form. It is the most common cause of chronic anemia among affected individuals. Hemoglobinopathy is an inherited blood disorder arising from mutations in globin genes and is broadly categorized into those involving structural changes that produce abnormal hemoglobin variants or defects in globin chain production. This review aims to evaluate the risk factors and outcomes of pulmonary hypertension among individuals with hemoglobinopathies. A search was conducted on PubMed and Google Scholar databases from inception to April 30, 2025. In total, 1,825 articles were synthesized, of which 13 were included in the final qualitative analysis and data extraction. We included English-language original articles published in peer-reviewed journals that reported the risk factors and outcomes of pulmonary hypertension in patients of any age and gender diagnosed with any type of hemoglobinopathy. This review synthesized 13 articles from 10 countries. A total of 2,873 individuals were diagnosed with hemoglobinopathy (1,031 (36%) with sickle cell disease and 1,842 (64%) with β-thalassemia), and 472 were diagnosed with pulmonary hypertension. Among those with pulmonary hypertension, 289 (61%) had sickle cell disease, while 183 (39%) had β-thalassemia. Older age (>40 years), a history of splenectomy, a hemoglobin level of <8 g/dL, frequent blood transfusions, frequent hospitalization for vaso-occlusive crisis, and β-thalassemia were associated with pulmonary hypertension. Some laboratory parameters (serum creatinine, reticulocyte, albumin, nucleated red blood cells, globulin, cell-free hemoglobin, N-terminal pro-B-type natriuretic peptide, high-sensitivity C-reactive protein, soluble vascular cell adhesion molecule, platelet, lactate dehydrogenase) were associated with pulmonary hypertension. Overall, the mortality rate was 27 (10%), with respiratory failure, sudden death, and cor pulmonale as the common causes of mortality. Early recognition and risk stratification for pulmonary hypertension must become integral components of hemoglobinopathy care, particularly in adult patients and those with high-risk profiles. Establishment of a standardized treatment guideline and optimizing the use of disease-modifying therapies, such as hydroxyurea and iron chelators, and exploring novel pharmacologic strategies (endothelin receptor antagonist, phosphodiesterase type 5 inhibitors), may hold promise for altering the trajectory of pulmonary hypertension in this vulnerable group. Our findings confirm that pulmonary hypertension is not only a prevalent complication but also a serious prognostic marker associated with increased morbidity and mortality in this population.

## Introduction and background

Hemoglobinopathies refer to a diverse group of inherited blood disorders and constitute the most prevalent monogenic disorders worldwide [1,2]. Hemoglobinopathies arise from mutations in globin genes and are broadly categorized into the following two main types: those involving structural changes that produce abnormal hemoglobin variants, and those involving defects in globin chain production, where one or more types of globin chains are either partially or completely absent [3]. In some cases, both structural abnormalities and synthesis defects may occur together. The combination of thalassemia variants with different structural hemoglobin variants results in a diverse spectrum of disorders, each with varying degrees of clinical severity [2].

Structural hemoglobinopathies are typically caused by point mutations, small insertions, or deletions in the coding regions of globin genes, resulting in the substitution of amino acids within the hemoglobin protein chain [3]. The most common structural hemoglobin variants include HbS, HbE, and HbC [4]. Thalassemias result from a decreased or absent production of one or more types of globin chains. These excess chains are unstable and tend to precipitate, leading to damage to the red blood cell membrane and premature destruction of the cells. Thalassemias are classified based on which specific globin chain production is impaired: α, β, γ, δ, δβ, γδβ [3].

While hemoglobinopathies were historically confined to certain geographical regions and populations, such as β-thalassemia in the Mediterranean, the Middle East, and the far East, and sickle cell disease in Sub-Saharan Africa and African American communities, they are now found globally due to increased migration to Western countries [1].

Despite variability in clinical presentation, hemoglobinopathies are typically severe, especially in individuals with homozygous forms. These patients often face reduced life expectancy, multiple organ complications, frequent hospital admissions, and the need for lifelong care, resulting in a substantial medical and socioeconomic burden [1]. Cardiovascular complications are among the main causes of illness and death in individuals with hemoglobinopathies [1]. Pulmonary hypertension is a common complication among hemoglobinopathies and is associated with an increased risk of early mortality [5]. According to the 6th World Symposium on Pulmonary Hypertension, pulmonary hypertension is defined as a mean pulmonary artery pressure of ≥20 mmHg at rest, as measured by right heart catheterization [6], or a tricuspid regurgitation velocity (TRV) of >2.5 m/s with the aid of transthoracic Doppler echocardiography [7]. The majority prefers the non-invasive screening method (TRV) over the invasive (mean pulmonary arterial pressure) due to convenience and lack of injury during the procedure. Pulmonary hypertension has become a major contributor to illness and death among patients with hemoglobinopathies and chronic hemolytic anemia [8]. Hemoglobinopathies are of public health concern, accounting for 71% of 229 countries, with these 71% of countries accounting for 89% of all births worldwide. About 330,000 infants are affected annually, of which 83% have sickle cell disorder and 17% have thalassemia. Hemoglobin disorders account for approximately 3.4% of under-five mortality. Globally, about 7% of pregnant women are carriers of β or α zero thalassemia or hemoglobin S, C, D Punjab, or E [9].

According to Farmakis and Aessopos, hemoglobinopathy is associated with a chronic hemolysis that leads to nitric oxide depletion, arginine catabolism, and endogenous nitric oxide synthesis inhibition. This enhances platelet activation and increases endothelin-1 release, which results in the development of vasculopathy, characterized by endothelial dysfunction, increased vascular tone, inflammation, hypercoagulability, and, eventually, vascular remodelling and destruction of pulmonary vasculature, which ultimately results in pulmonary hypertension [1]. The common symptoms of pulmonary hypertension in hemoglobinopathies include exertional dyspnea, fatigue, exercise intolerance, signs of right heart failure (peripheral edema and ascites), and jaundice [10]. The diagnosis of hemoglobinopathy requires a red blood cell count with erythrocyte indices and a hemoglobin test with hemoglobin electrophoresis and/or chromatography [4].

This review aims to evaluate the risk factors and outcomes of pulmonary hypertension among individuals with hemoglobinopathies.

## Review

Methodology

This systematic review followed the Preferred Reporting Items for Systematic Reviews and Meta-Analyses (PRISMA) guidelines [11]. This study protocol was registered with Prospero (registration ID: CRD420251039454).

Inclusion Criteria

We included original articles published in peer-reviewed journals and written in the English language that reported the risk factors and/or outcomes of pulmonary hypertension in patients of any age and gender diagnosed with any hemoglobinopathy.

Exclusion Criteria

We excluded comments, reports, letters, surveys, abstracts, case series, editorials, systematic reviews, meta-analyses, articles not written in the English language, and studies including patients without hemoglobinopathy.

Search Strategy

A comprehensive search was done on PubMed and Google Scholar databases from inception to April 30, 2025, using the following search phrases on the searched databases: (pulmonary hypertension) AND (Hemoglobinopathies), “pulmonary hypertension” “Hemoglobinopathies.”

Eligible articles, duplicate articles, titles, and abstracts were screened by four co-authors against the predefined eligibility criteria using the Rayyan reference manager [12]. After the abstract screening, eligible articles were subjected to a full-text screen. Disagreements were discussed among reviewers. In the case of no resolution, an appeal was made to another reviewer.

Data from the eligible articles was extracted by four coauthors independently and recorded on a Google spreadsheet. The variables included author(s) name, country, study year, study design, sample size, follow-up, pulmonary hypertension diagnostic tool, type of hemoglobinopathy, gender, mean age, current medication intake, blood transfusion, frequency of crisis, mean hematocrit, complications, risk factors, and outcomes. Eligible articles were assessed for risk of bias using the Joanna-Briggs Institute (JBI) critical appraisal tool for case-control, cross-sectional, and cohort studies, assessing the trustworthiness, relevance, and results of published papers. The purpose of this appraisal was to assess the methodological quality of the study and to determine the extent to which the study addressed the possibility of bias in its design, conduct, and analysis. Articles were assessed with a yes, no, not clear, and not applicable [13]. The search strategy and the JBI critical appraisal tool findings are presented in the Appendices.

Results

**Figure 1:**
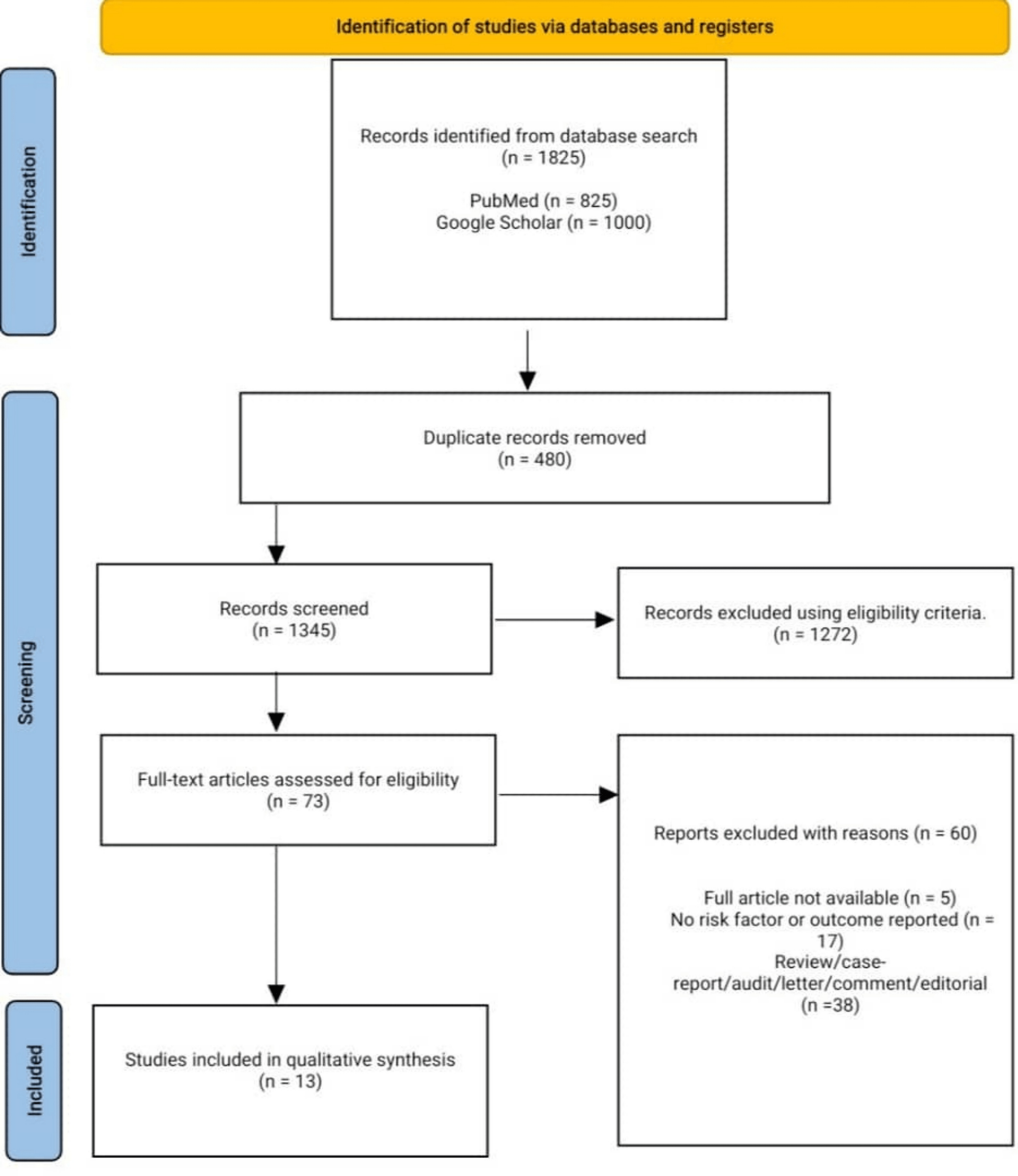
Preferred Reporting Items for Systematic Reviews and Meta-Analyses (PRISMA) flow diagram.

Our database search returned 1,825 articles, which were screened for duplication, and duplicated articles were removed. After removing duplicates, 1,345 articles were screened for the title and abstract, and 1,272 were eliminated following our predefined eligibility criteria. A total of 73 articles underwent a full-text screening, and 13 were included in the final qualitative analysis and data extraction. In total, 60 articles were excluded due to the unavailability of the full text, the lack of risk factors or outcomes of pulmonary hypertension among individuals living with hemoglobinopathies, case reports, comments, editorials, abstracts, and reviews. Figure 1 shows the PRISMA flow diagram.

Study Characteristics

A total of 2,873 diagnosed hemoglobinopathy patients were included in this review. Overall, 472 patients were diagnosed with pulmonary hypertension, either with the use of echocardiography or right heart catheterization. Studies originated from 10 countries (the United States, Nigeria, Thailand, Italy, Brazil, Egypt, Iran, Oman, Lebanon, and the United Arab Emirates), with the United States contributing the most articles (five), followed by Thailand (three), Nigeria (two), and Italy (two). Of the total population diagnosed with hemoglobinopathy, 36% (1,031) were living with sickle cell disease, and 61% (289) had pulmonary hypertension. Further, 64% (1,842) of the remaining population was diagnosed with thalassemia, and 39% (183) had pulmonary hypertension. The study period was from 2001 to 2022, and the mean age ranged from 18 to 48 years. Overall, 117 male and 169 female patients with hemoglobinopathy were diagnosed with pulmonary hypertension. Table 1 presents further demographic data.

**Table 1:** Study demographics. SCD: sickle cell disease; N/A: not available

Author	Year	Country	Hemoglobinopathy	Sample size	Pulmonary hypertension	Mean age	Male	Female
Taylor et al. [5]	2008	USA	SCD	260	95	N/A	N/A	N/A
Ashley-Koch et al. [14]	2007	USA	SCD	111	44	N/A	N/A	N/A
Inthawong et al. [15]	2014	Thailand	Thalassemia	76	7	21	3	4
Karimi et al. [16]	2011	Egypt, Iran, Oman, Italy, Lebanon, UAE	Thalassemia	128	64	37	28	36
Castro et al. [17]	2001	USA	SCD	34	20	37	6	14
Atichartakarn et al. [18]	2014	Thailand	Thalassemia	110	41	29	25	16
Odeyemi et al. [19]	2022	Nigeria	SCD	113	7	18	1	6
Lobo et al. [20]	2010	Brazil	SCD	125	43	34	19	24
Derchi et al. [21]	2013	Italy	Thalassemia	1,309	47	48	18	29
Peter et al. [22]	2016	Nigeria	SCD	100	22	7	N/A	N/A
Teawtrakul et al. [23]	2013	Thailand	Thalassemia	219	24	37	15	9
De Castro et al. [24]	2005	USA	SCD	125	42	44	20	22
Gordeuk et al. [25]	2005	USA	SCD	163	16	43	7	9

Among the included articles, two studies did not mention the mean age [5,14], while three studies did not mention the number of male and female participants [5,14,22]. The largest sample size was 1,309, while 95 were among those diagnosed with pulmonary hypertension. The smallest sample size was 34, while seven were among those diagnosed with pulmonary hypertension. The study designs included case-control [5,14-18], cross-sectional [19-23], and cohort studies [24,25]. The mean body mass index recorded among the three studies ranged from 18 to 20 kg/m^2^ [18,20,25]. Some studies reported intake of some medications, and 49 patients with pulmonary hypertension were currently on hydroxyurea [16,19,20,24], 65 on an iron chelator [16,18], five on sildenafil [23], and eight on prostacyclin infusion [17]. A total of 151 (27%) patients received blood transfusions, and the mean hematocrit from the included studies ranged from 21.6% to 27.0%. Some studies recorded the presence of complications of hemoglobinopathy among patients, which included 6% (21) strokes, 19% (25) osteomyelitis, 5% (11) priapism, 30 (13%) acute chest syndrome, 69 (31%) acute painful crises, 1 (0.8%) seizures, 11 (9%) leg ulcers, 6 (5%) all cardiovascular events, and 72 (72%) frequent hospitalizations.

Risk Factors and Outcomes

The included studies used echocardiography and right heart catheterization to diagnose pulmonary hypertension. However, the majority of the studies used echocardiography (12 studies), four studies used echocardiography and right heart catheterization, while one study used right heart catheterization alone. The average TRV >3.0 was recorded among hemoglobinopathy patients diagnosed with pulmonary hypertension. The most reported risk factors among the included studies were age >40 years [14,19-21], splenectomy [15,16,18,21,23], hemoglobin level <8 g/dL [14,19-21,24], frequent blood transfusions [15,22], frequent hospitalizations due to vaso-occlusive crises [5,22], elevated blood pressure [19,25], and β-thalassemia hemoglobinopathy [21,23]. Aticharkarn et al. [18] reported some laboratory findings predisposing an individual with hemoglobinopathy to developing pulmonary hypertension. The majority of the studies did not indicate the number of patients with a specific predisposing factor to pulmonary hypertension. Details of the risk factors are presented in Table 2.

**Table 2:** Risk factors of pulmonary hypertension. BMI: body mass index; SpO_2_: peripheral oxygen saturation; NT-pro-BNP: N-terminal pro-B-type natriuretic peptide; hs-CRP: high-sensitivity C-reactive protein; sVCAM: soluble vascular cell adhesion molecule; LDH: lactate dehydrogenase; N/A: not available; n: total number of individuals with risk factors; N: total population size in which the risk factor of interest was mentioned

Risk factors	Number of patients (n)	Percentage (n/N)
Splenectomy	147	80%
β-thalassemia	52	73%
Cardiomegaly	21	51%
Single-nucleotide polymorphism	5	11%
Blood pressure >140/90 mmHg	16	100%
Age >40 years	N/A	N/A
Hemoglobin <8 g/dL	N/A	N/A
BMI >20 kg/m^2^	N/A	N/A
Hospitalization for acute crisis	N/A	N/A
Frequent blood transfusions	N/A	N/A
Thromboembolic events	N/A	N/A
Non-hydroxyurea intake	N/A	N/A
Frequent vaso-occlusive hospitalization	N/A	N/A
SpO_2_ <93%	N/A	N/A
Serum creatinine >126 µmol/L	N/A	N/A
Reticulocyte >12%	N/A	N/A
Albumin <40 g/dL	15	37%
Nucleated RBC >354.2 × 10^6^/L	28	68%
Globulin >4.31g/dL	19	46%
Cell-free hemoglobin >3.0 g/dL	19	46%
NT-pro BNP > 6.5 pm	19	46%
hs-CRP >3.0 mg/L	12	29%
sVCAM >1,600 ng/mL	18	44%
Platelet >450 × 109/L	28	68%
LDH >200 U/L	21	51%

Only Castro et al., Lobo et al., and De Castro et al. [17,20,24] reported on mortality, highlighting that individuals diagnosed with pulmonary hypertension have an increased mortality rate. The overall mortality reported in the included study was 27 (10%), and Castro et al. and De Castro et al. reported a mean survival of 25.6 and 49 months, respectively. Castro et al. [17] reported respiratory failure, sudden cardiac death, and cor pulmonale as the cause of mortality. The outcome of pulmonary hypertension is illustrated in Table 3.

**Table 3:** Outcome of pulmonary hypertension among individuals with hemoglobinopathy. N/A: not available

Author	Outcome (morality)	Mean survival (months)	Follow-up (months)
Castro et al. [17]	11	25.6	23
Lobo et al. [20]	7	N/A	15
De Castro et al. [24]	9	49	62.5

Discussion

This systematic review provides a comprehensive synthesis of the current literature on pulmonary hypertension in individuals with hemoglobinopathies, specifically focusing on patients with sickle cell disease and thalassemia. By analyzing data from 13 studies conducted across 10 countries, this study draws attention to both the geographical diversity and clinical heterogeneity of hemoglobinopathy populations who develop pulmonary hypertension. The findings affirm the significant burden of pulmonary hypertension in this population, highlight multiple risk factors, and underscore the substantial mortality associated with the condition.

Study Demographics and Clinical Profile

A total of 2,873 individuals diagnosed with hemoglobinopathies were included in this review, of whom 472 (16.4%) were diagnosed with pulmonary hypertension through echocardiography or right heart catheterization. The distribution of cases across 10 countries, including the United States, Nigeria, Thailand, Brazil, and multiple Middle Eastern nations, underscores the global nature of the condition. However, regional disparities in study frequency and diagnostic reporting were evident. For instance, the United States contributed the highest number of studies, with five studies accounting for nearly one-third of the total sample. In contrast, regions with high burdens of hemoglobinopathies, particularly Sub-Saharan Africa, were underrepresented in the published literature, possibly due to limitations in diagnostic infrastructure or publication access [26,27].

Our findings reported that sickle cell disease accounted for 36% of the total hemoglobinopathies in the included study population, and made up 61% of those diagnosed with PH, reinforcing its stronger association with pulmonary hypertension compared to thalassemia. This finding aligns with prior studies that have reported pulmonary hypertension prevalence in sickle cell disease ranging from 20% to 30%, particularly when using echocardiographic criteria such as TRV >2.5-3.0 m/s [28,29]. In contrast, thalassemia, which comprised 64% of the study population, showed a lower relative frequency of pulmonary hypertension, with only 39% of pulmonary hypertension cases. This pattern is consistent with the distinct pathophysiological mechanisms underlying pulmonary hypertension in thalassemia, where splenectomy, chronic iron overload, and hypercoagulability play more prominent roles than the hemolysis-dominant pathway observed in sickle cell disease [30].

The mean age of the participants ranged from 18 to 48 years, which is reflective of the chronic nature of pulmonary hypertension and its emergence as a late complication of longstanding hemoglobinopathy [31]. Among the studies that reported sex-specific data, females appeared slightly more represented among pulmonary hypertension patients. However, this observation cannot be meaningfully interpreted without more rigorous sex-disaggregated analyses. The study designs included a mix of case-control, cross-sectional, and cohort studies, indicating varied methodological rigor.

Risk Factors, Diagnostic Challenges, and Treatment

The most frequently reported risk factors for pulmonary hypertension among hemoglobinopathy patients included advanced age (typically >40 years), a history of splenectomy, low hemoglobin levels (<8 g/dL), frequent hospitalizations due to vaso-occlusive crises, high blood pressure, and frequent blood transfusions. These findings mirror existing literature that has identified both chronic hemolytic anemia and hypercoagulable states as central pathways in the development of pulmonary hypertension in these patient populations [32,33]. Specifically, splenectomy was reported in 80% of pulmonary hypertension cases and was particularly common among patients with thalassemia. This is in line with findings from Karimi et al. [16], who described the role of the procedure in promoting platelet activation, endothelial damage, and pulmonary vascular remodeling [11].

Across the 13 studies, the diagnosis of pulmonary hypertension was predominantly made through echocardiography, with a TRV >3.0 m/s being the most common diagnostic threshold. Although right heart catheterization is considered the diagnostic gold standard, its use was limited, likely due to its invasiveness, availability constraints in certain regions, and or availability of experienced operators. This diagnostic preference, while practical, may contribute to the overestimation of pulmonary hypertension prevalence, as previously highlighted by Hayes et al. [32].

Among laboratory parameters, elevated levels of lactate dehydrogenase, cell-free hemoglobin, N-terminal pro-B-type natriuretic peptide, and soluble vascular cell adhesion molecule were frequently noted in pulmonary hypertension patients. These biomarkers are indicative of hemolysis-induced endothelial dysfunction, supporting the hemolytic vasculopathy hypothesis originally proposed by Kato et al. (2006) and Gladwin et al. (2004) [33,34]. Additional markers such as elevated platelet counts, nucleated red blood cells, and low albumin levels suggest that both inflammation and hypercoagulability further compound the risk of pulmonary hypertension [33,34]. However, the clinical utility of these biomarkers as prognostic or screening tools requires further validation in prospective studies.

Interestingly, despite the frequent discussion of hydroxyurea in sickle cell disease management, only 49 patients across four studies were reported to be receiving it at the time of pulmonary hypertension diagnosis. Hydroxyurea has been associated with reduced hemolysis and improved endothelial function [35], suggesting a potentially protective role against pulmonary hypertension that was not sufficiently explored in the included studies. Similarly, data on the use of sildenafil and prostacyclin analogs were limited, even though these therapies are established treatments in other forms of pulmonary arterial hypertension, suggesting a therapeutic gap in this population [36,37]. The underutilization of these medications might be due to the lack of standardized treatment guidelines for pulmonary hypertension, the unavailability of these drugs, or the lack of knowledge of their use.

Outcomes and Prognostic Insights of Pulmonary Hypertension

The outcome data from the reviewed studies paint a sobering picture. Among the 472 pulmonary hypertension patients, 27 deaths were recorded, resulting in an overall mortality rate of 10%. The majority of deaths were attributed to respiratory failure, sudden cardiac death, and cor pulmonale, complications that have been previously described as terminal endpoints in the course of pulmonary hypertension among sickle cell disease and thalassemia patients [17,24]. Mean survival durations were 25.6 months and 49 months in the two studies that provided follow-up data, further emphasizing the prognostic significance of pulmonary hypertension in these populations.

These findings are congruent with longitudinal studies such as that by Machado et al. [38], who demonstrated that elevated TRV was independently associated with increased mortality in sickle cell disease. Similarly, Ataga et al. found that SCD patients with echocardiographic evidence of pulmonary hypertension had a relative risk of death over 10 times higher than those without [29]. Given the association between pulmonary hypertension and early mortality, its early identification should be prioritized in routine care protocols, especially for older patients, those with prior splenectomy, or with frequent vaso-occlusive episodes.

Although some patients were reported to be receiving disease-modifying therapies such as hydroxyurea or iron chelators, there was no consistent association between therapy and outcome due to poor reporting and study design limitations. As such, the effectiveness of these interventions in altering the natural history of pulmonary hypertension in hemoglobinopathies remains an area in urgent need of randomized controlled trials.

Strengths and limitations

This review offers several notable strengths that enhance its relevance and contribution to the understanding of pulmonary hypertension in patients with hemoglobinopathies. First, it is one of the few syntheses to include studies across multiple geographic regions, encompassing low-, middle-, and high-income countries, thereby capturing the global diversity of patient populations with sickle cell disease and thalassemia. This global scope improves the generalizability of the findings and enables cross-regional comparisons that are often lacking in single-center or regional studies.

Another key strength is the relatively large pooled sample size of 2,873 patients, which provides a robust platform to explore the frequency, risk factors, and outcomes associated with pulmonary hypertension in hemoglobinopathies. Importantly, the study includes both pediatric and adult populations, widening the age spectrum and reflecting the chronic, progressive nature of the disease. In addition, the structured data extraction and synthesis approach, particularly the categorization of risk factors into clinical, laboratory, and demographic domains, allows for a multi-dimensional understanding of pulmonary hypertension development. Furthermore, the analysis did not restrict itself to a single study design; instead, it drew from case-control, cross-sectional, and cohort studies, which collectively contribute a broader evidence base for understanding this complex pathology.

However, several limitations must also be acknowledged. There are fewer studies from high hemoglobinopathy burden regions such as Sub-Saharan Africa and South Asia. The exclusion of non-English-language publications may limit the number of included studies. While echocardiography using TRV >3.0 m/s was commonly employed, only a minority of studies used the gold-standard right heart catheterization for confirmation. This raises the possibility of misclassification bias, with potential overestimation of pulmonary hypertension prevalence due to the relatively low specificity of echocardiographic methods in hemoglobinopathy populations.

Another limitation lies in the lack of a standardized treatment guideline for individuals with hemoglobinopathy who have developed pulmonary hypertension and inconsistent reporting of clinical and demographic variables. Key data such as age, sex, body mass index, and treatment regimens (e.g., hydroxyurea, iron chelators) were missing or incomplete in several studies. This limited the ability to perform subgroup analyses or adjust for confounding factors. Additionally, most included studies were observational and thus prone to selection bias, information bias, and inability to establish causal relationships between risk factors and pulmonary hypertension outcomes.

The time range of the included studies, spanning from 2001 to 2022, also introduces temporal heterogeneity, as changes in clinical management (e.g., wider use of hydroxyurea, improved transfusion safety, or earlier diagnosis of pulmonary hypertension) may have influenced outcomes. The absence of standardized follow-up periods further constrained the assessment of survival outcomes and treatment efficacy.

Another challenge encountered in this review was the lack of harmonized definitions and thresholds for laboratory markers, such as lactate dehydrogenase, N-terminal pro-B-type natriuretic peptide, and reticulocyte counts, which impedes direct comparisons and meta-analytical approaches. Furthermore, many studies failed to report the number of patients exposed to individual risk factors, limiting quantitative synthesis of attributable risk.

Finally, although the inclusion of both sickle cell disease and thalassemia expands the scope of the review, these conditions differ significantly in pathophysiology, clinical course, and management. As such, pooling their data, while useful for overarching insights, may obscure disease-specific patterns that could be better addressed in separate analyses.

## Conclusions

Early recognition and risk stratification for pulmonary hypertension must become integral components of hemoglobinopathy care, particularly in adult patients and those with high-risk profiles. Establishing standardized treatment guidelines, optimizing the use of disease-modifying therapies, such as hydroxyurea and iron chelators, and exploring novel pharmacologic strategies (endothelin receptor antagonist, phosphodiesterase-5 inhibitors) may hold promise for altering the trajectory of pulmonary hypertension in this vulnerable group. Our findings confirm that pulmonary hypertension is not only a prevalent complication but also a serious prognostic marker associated with increased morbidity and mortality in this population.

## Appendices

**Table 4:** Search strategy.

Search query	Database	Outcome
(pulmonary hypertension) AND (Hemoglobinopathies)	PubMed	825
“pulmonary hypertension” “Hemoglobinopathies”	Google Scholar	1,000

**Table 5:** Joanna-Briggs Institute (JBI) critical appraisal tool for case-control studies.

Checklist question	Taylor et al. [5]	Ashley-Koch et al. [14]	Inthawong et al. [15]	Karimi et al. [16]	Castro et al. [17]	Atichartakarn et al. [18]
Were the groups comparable other than the presence of disease in cases or the absence of disease in controls?	Yes	Yes	Yes	Yes	Yes	Yes
Were cases and controls matched appropriately?	Yes	Yes	Yes	Yes	Yes	Yes
Were the same criteria used for identification of cases and controls?	Yes	Yes	Yes	Yes	Yes	Yes
Was exposure measured in a standard, valid and reliable way?	Yes	Yes	Yes	Yes	Yes	Yes
Was exposure measured in the same way for cases and controls?	Yes	Yes	Yes	Yes	Yes	Yes
Were confounding factors identified?	Yes	Yes	Yes	Yes	Yes	Yes
Were strategies to deal with confounding factors stated?	Yes	Yes	Yes	Yes	Yes	Yes
Were outcomes assessed in a standard, valid and reliable way for cases and controls?	Yes	Yes	Yes	Yes	Yes	Yes
Was the exposure period of interest long enough to be meaningful?	Yes	Yes	Yes	Yes	Yes	Yes
Was appropriate statistical analysis used?	Yes	Yes	Yes	Yes	Yes	Yes

**Table 6:** Joanna-Briggs Institute (JBI) critical appraisal tool for cross-sectional studies.

Checklist question	Odeyemi et al. [19]	Lobo et al. [20]	Derchi et al. [21]	Peter et al. [22]	Teawtrakul et al. [23]
Were the criteria for inclusion in the sample clearly defined?	Yes	Yes	Yes	Yes	Yes
Were the study subjects and the setting described in detail?	Yes	Yes	Yes	Yes	Yes
Was the exposure measured in a valid and reliable way?	Yes	Yes	Yes	Yes	Yes
Were objective, standard criteria used for measurement of the condition?	Yes	Yes	Yes	Yes	Yes
Were confounding factors identified?	Yes	Yes	Yes	Yes	Yes
Were strategies to deal with confounding factors stated?	Yes	Yes	Yes	Yes	Yes
Were the outcomes measured in a valid and reliable way?	Yes	Yes	Yes	Yes	Yes
Was appropriate statistical analysis used?	Yes	Yes	Yes	Yes	Yes

**Table 7:** Joanna-Briggs Institute (JBI) critical appraisal tool for cohort studies.

Checklist question	De Castro et al. [24]	Gordeuk et al. [25]
Were the two groups similar and recruited from the same population?	Yes	Yes
Were the exposures measured similarly to assign people to both exposed and unexposed groups?	Yes	Yes
Was the exposure measured in a valid and reliable way?	Yes	Yes
Were confounding factors identified?	Yes	Yes
Were strategies to deal with confounding factors stated?	Yes	Yes
Were the groups/participants free of the outcome at the start of the study (or at the moment of exposure)?	Yes	Yes
Were the outcomes measured in a valid and reliable way?	Yes	Yes
Was the follow up time reported and sufficient to be long enough for outcomes to occur?	Yes	No
Was follow up complete, and if not, were the reasons to loss to follow up described and explored?	Yes	No
Were strategies to address incomplete follow up utilized?	No	No
Was appropriate statistical analysis used?	Yes	Yes
